# The International Medical Annual and Practitioner’s Index

**Published:** 1895-04

**Authors:** 


### BOOK NOTICES.

The International Medical Annual and Practitioner's Index. A work of reference for medical practitioners. New York: E. B. Treat, 5 Cooper Union. Chicago: 199 Clark Street. 1895. Price, $2.75.

The thirteenth annual issue of this valuable publication is now before us. It has been reviewed in this journal regularly every year, so that our readers must be quite familiar with its qualities.

As has been stated before, it contains the latest developments in every branch of medical science during the past year.

Everything is arranged in alphabetical order and the names of the subject-matter placed in large letters at the top of the page. In addition it has a thorough and most minute index to every important statement in the book.

The present issue treats very extensively of the subject of eye-strain, especially in children going to school. This important question is illustrated by photographs, showing the positions of pupils in schools when reading and writing, demonstrating the right and the wrong method of seating the pupils, with regard to the light and their own stature. This is highly important to every physician.

A considerable space is devoted to the anti-toxine treatment of diphtheria. This is exhaustively discussed and includes everything now known.

The most remarkable developments in sanitary science, such as the management of sewers, are well shown and are exceedingly interesting and important.

The whole work is a magaziue of the most valuable information, arranged in the most ready form for instant reference.

